# Why do people resist algorithms? From the perspective of short video usage motivations

**DOI:** 10.3389/fpsyg.2022.941640

**Published:** 2022-08-24

**Authors:** Xinzhou Xie, Yan Du, Qiyu Bai

**Affiliations:** School of New Media, Peking University, Beijing, China

**Keywords:** algorithms, algorithmic resistance, affordance, short video, motivation

## Abstract

Algorithms embedded in media applications increasingly influence individuals’ media practice and behavioral decisions. However, it is also important to consider how the influence of such algorithms can be resisted. Few studies have explored the resistant outcomes of the interactions with algorithms. Based on an affordance perspective, this study constructed a formation framework of algorithmic resistance in the context of short videos in China. Survey responses from 2,000 short video users to test the model. Exploratory factor analysis, confirmatory factor analysis, and structural equation modeling were used for data analysis. The findings reveal two types of “moderate” resistance: avoidance and obfuscation. Specific needs, such as the motivations of peeking and escapism, are significantly related to perceived algorithmic affordance, which, in turn, encourages the tactics of avoidant and obfuscated resistance. The results provide new insights into the potential formation mechanisms of algorithmic resistance. The forms of resistance highlighted in the paper evolve alongside algorithms and have significant practical implications for users and platforms.

## Introduction

Algorithms are an essential part of Internet infrastructure and have reshaped reality, especially media life (Deuze, 2011). Algorithms have transformed the presentation of information and interaction by aggregating, filtering, recommending, and rating (Kitchin, 2017; Dogruel et al., 2020). Because the interactions between algorithms and users can be considered a socio-technological system with recursive relationships (Gillespie, 2014; Willson, 2017), insights into how users perceive and interact with these systems are essential.

Much of past research on algorithms has addressed the “transformative effects” (Kitchin, 2017) of algorithms as powerful information agents, as well as the potential risks and consequences (Neyland and Möllers, 2017; Dogruel et al., 2020; Gran et al., 2020). A few scholars have started to focus on users’ perceptions and behavior of recommendation algorithms. The previous study found that users’ awareness of algorithms was low and their level of understanding varied widely (Eslami et al., 2015; Rader and Gray, 2015). Due to the “black box” attributes of algorithms, the research method has been a challenge for scholars in the social sciences. But without empirical evidence from the user perspective, it is also impossible to develop effective policies on algorithms (Latzer and Festic, 2019). Prior studies on users’ perceptions and behaviors about algorithms were mostly carried out in qualitative methods. For example, Bucher (2017) studied users’ awareness and experience of algorithms in daily life through tweets and interviews. Bishop (2019) adopts an ethnographic approach to study the formation of beauty vloggers’ algorithmic knowledge and how it guides content production.

In terms of research content, previous studies focused on algorithms in social media News Feed. Scholars have proposed some concepts, such as algorithmic imaginary, algorithmic gossip, algorithmic skills, and algorithmic knowledge, to describe users’ perceptions and understanding of algorithms (Bucher, 2017; Bishop, 2019; Hargittai et al., 2020). Folk theories have also been developed to explain how users’ perceptions guide practice (DeVito et al., 2018). And in recent years, research has gradually expanded into more areas, such as video sites, product content, and advertising. Some papers analyzed user behaviors when confronted with algorithms and new types of advertising in videos (Belanche et al., 2017a,b). And several studies found that influencers, especially vloggers, have a good understanding of algorithms and prefer to use algorithms to increase their popularity and build and govern audiences (Cotter, 2019; Mac Donald, 2021).

While critical work is coming out in this area, there are also some gaps. These findings explained the actions of digital subjection when encountering algorithms yet lacked attention to resistance. The new information systems, like recommendation algorithms and skippable advertising, not only change the way information is presented, but also empower the user (Belanche et al., 2019, 2020). Users’ active roles are reflected both in the ability to use the algorithms to achieve their goals and in the ability to maintain critical thinking and resist. However, only a few studies have theoretically analyzed the possibilities of user resistance, while in-depth empirical studies are inadequate. Velkova and Kaun (2021) emphasized that as algorithms play a predominant role in media power, it is becoming increasingly critical to deliberate how and to what extent their power can be resisted. Furthermore, most research does not extend beyond users in the West, leaving issues for platforms and users in other cultural settings as a gap in the literature.

To fill these gaps, this paper studies the formation mechanism of algorithmic resistance in the context of short videos in China. Specifically, the research aims to resolve how people resist algorithms and what factors would trigger the tactics of resistance. This study contributes in three areas. First, the research provides new insights into the potential formation mechanisms of algorithmic resistance by introducing affordance perspectives. The paper constructed a structural equation model to explain how the specific needs of users trigger algorithmic resistance through the mediating role of perceived explainability of algorithms, which empirically supports the formation framework of algorithmic resistance. Second, the paper has extended the research on algorithmic awareness and behavior to the context of short video platforms. Past research mostly touched on Facebook, YouTube, and Instagram. However, short video platforms (like Douyin and Kuaishou), which are driven by algorithms, have become one of the most popular social media platforms, especially in China. The research fully considers the impact of users’ specific intention of using short videos on their perception and understanding of algorithms, as well as resistance behavior. Third, this study emphasizes the tactics of avoidance and obfuscation, which expands the range of possibilities for actions on algorithms. These findings could have important implications for enhancing the autonomy of user content curation, optimizing algorithms, and promoting the sustainable development of social media ecosystems.

## Theoretical background and hypotheses

### Affordance perspectives and algorithmic affordance

As a complex social technology system, algorithms have been embedded in all subsystems of society. The research on algorithm issues in the field of social sciences can be classified as the research on the relationship between technology and society. The research in this field is common in two theoretical value orientations, one is technological determinism, and the other is social constructivism. Especially in the face of the application brought by the new technological revolution, the proposition of technological determinism is more powerful. For example, in the early stage, due to the black box nature of algorithms (Pasquale, 2015), scholars mostly used the viewpoint of technological determinism to discuss the strong impact, risks, and challenges of algorithms on society. But only from the perspective of technological determinism, we cannot explain how algorithms perform specific tasks in various social practices, which involves the sociality of algorithms. The viewpoint of social constructivism began to appear. Social constructivism emphasizes the subjective role of human beings. However, some scholars have found that it is difficult to fully explain the use of algorithms from the perspective of constructivism. The role of people is also limited, and their behavior is affected by many factors. Thus, the concept of affordances is attractive for communication researchers because it suggests that neither materiality nor a constructivist view is sufficient to explain technology use (Leonardi and Barley, 2008), and advocates focusing on relational actions that occur among people and technologies (Faraj and Azad, 2012).

Gibson (1979), the ecological psychologist who first proposed the concept of “affordance,” held a relational view of animals and the environment, exploring what affordances and actionable possibilities the environment provides the animal. Subsequent scholars have since reinterpreted the concept prescriptively in human-computer interaction (Norman, 1988; Kuhn, 2012), to reflect a valuable approach in the design of software.

Norman (1988) defined affordances as “the perceived and actual properties of an object, primarily those fundamental properties that determine how the thing could be used.” Given the focus on interface design, Norman abstracted the concept of “perceived affordance,” referring to the range of possible actions perceived by the user of an object. Norman considered that a good designer should make an effort to reduce the gap between design affordances and perceived affordances, and focus on the users’ needs and cognitive models (Norman, 1993). His approach inspired later scholars to empirically research technology affordance from the perspective of users. From this viewpoint, affordance was related to the characteristics of technical functions and linked to the users’ intention, perception, and understanding. The affordance perspective provides a framework that describes the many-sided relational structure between a technology and the users that allows or restricts possible behavioral outcomes in a particular context. The framework helps interpret the mutuality between the users, the material features of the technologies, and the situational nature of use (Evans et al., 2016).

The perspective of affordance is appealing to communication scholars because it suggests that any one-side view (materiality or constructivist) is insufficient to explain the use of technologies (Leonardi and Barley, 2008). The approach advocates a focus on the relational actions occurring between humans and technologies. Thus, media scholars introduced affordance perspectives into the studies of human and artificial intelligence (AI) interaction to answer the questions, such as “What can AI offer to users?” “How can AI allow users action, and how can we use AI for our particular needs?” (Jakesch et al., 2019; Flanagin, 2020; Sundar, 2020).

Shin and Park (2019) proposed an operational definition for the concept of “algorithmic affordance.” They referred to the possibilities of actions that people perceive concerning features in algorithms, based on four dimensions – fairness, accountability, transparency, and explainability (Shin, 2020). Users have limited direct evidence for the first three dimensions. Because few platforms officially disclose information about the training set and models of recommendation algorithms (Cotter, 2019). Explainability of an algorithm could be referred to as the ability to explain how an algorithm works and what range of potential outcomes it has offered (Barredo Arrieta et al., 2020). Users can understand and explain the outputs or procedures of the algorithms through experimentation and adaptation to the interface (Leonardi et al., 2011). There were also many folk theories (Ytre-Arne and Moe, 2021) about algorithms on how recommendation algorithms function through recursive loops of the interaction between users and algorithms (Gillespie, 2014). This means that the recommendation systems of a social media platform provide the possibilities to be understood. Furthermore, Shin (2021) proved that the explainability of algorithms was the premise of perceived fairness, perceived transparency, and accountability. Thus, explainability is the most crucial element (Ehsan and Riedl, 2019; Barredo Arrieta et al., 2020).

### Particular needs and perception: Trigger process of the sensing algorithm

A small number of studies have investigated Internet user awareness of algorithms (Bucher, 2017; Proferes, 2017; Klawitter and Hargittai, 2018). For example, a laboratory study recruited 40 Facebook users to examine perceptions of Facebook’s News Feed algorithms and found that the bulk of participants (62.5%) were not aware of the algorithms and did not understand them (Eslami et al., 2015). In contrast to Eslami’s results, other studies indicate that most survey respondents perceive that they are aware of algorithms, although the level of awareness varies (Rader and Gray, 2015; Gran et al., 2020). Despite contradictory findings, these studies raise an important question: in what usage scenarios do people become aware of and perceive algorithms? What are the predictors of this awareness?

The affordance approach emphasizes the multifaceted relationship between individuals’ goals, the properties of technology, and the context in which the technology is used (Evans et al., 2016). Social media research has long proposed that social media use is goal-driven to meet individual needs (Papacharissi and Mendelson, 2011). The short video is an important social media driven by algorithms, which has developed rapidly in recent years. Algorithms have transformed the presentation of information and interaction by aggregating, filtering, recommending, and rating (Kitchin, 2017; Dogruel et al., 2020). Therefore, compared with other social media platforms, the change of short videos in information presentation and reception is more attractive to users. Ordinary users mainly use short videos for entertainment, escapism, and peeking (Fayard and Weeks, 2014; Omar and Dequan, 2020; Rach and Peter, 2021). Although self-presentation is also one of the motivations for people to use short videos, in this context, the goals of ordinary users and vloggers are not different. Thus, this study focuses on these goals in using short videos entertainment, escapism, and peeking. For example, TikTok is regarded as the go-to app for escapism (Rach and Peter, 2021). Based on the affordance perspective, Norman (1988) believed that the perceived affordance was subject to the mental models of users. Users can perceive a range of action possibilities from technology, but the nature and level of the perceived affordance depend on the goals and usage scenarios of users (Fayard and Weeks, 2014). Thus, based on affordance theory, this study infers those goals for short video use will facilitate the perceived explainability of recommendation algorithms. The study hypotheses were as follows:

*H1*: Entertainment is positively related to perceived explainability of algorithms.

*H2*: Peeking is positively related to perceived explainability of algorithms.

*H3*: Escapism is positively related to perceived explainability of algorithms.

### Algorithmic resistance: User heuristics of algorithmic affordance

Many researchers have concentrated on the negative impacts of algorithms, such as ethical problems related to invisibility and accountability (Bucher, 2012; Kitchin, 2017; Ananny and Crawford, 2018). However, limited attention has been paid to the possibility of users resisting algorithmic power. Unlike the conventional understanding of resistance—often regarded as an organized collective action – algorithmic resistance is unorganized and individual. It implies accommodation with the systems rather than remaining mutually exclusive (Scott, 2008). Users are not passive observers in this process. Velkova and Kaun (2021) revealed a progressive role users played in reshaping the operation of algorithms. People begin to develop tactics of resistance through alternative uses. Ettlinger (2018) offered a taxonomy of resistance, including productive resistance, avoidance, and disruption or obfuscation. For ordinary Internet platform users, the “threshold” of productive resistance, such as hacking, platform cooperatives, and “cloud protesting” (Ettlinger, 2018), is too high to be widely popular. Thus, this study focused on two other tactics of resistance. Avoidance is a mode of strategy involving complete or resistance partially withdrawal from digital platforms (Ettlinger, 2018). It refers specifically to the behavioral outcome where users do not interact with the platforms in this study. Another way of algorithmic resistance aims to obfuscate the processes of algorithms through “tricking” algorithms, for example, producing false behavioral data to confuse the algorithms, ensuring that user preferences remain unidentified (Brunton and Nissenbaum, 2015; Velkova and Kaun, 2021).

The theoretical framework of affordance suggests that users’ perception and understanding of technologies enable the possible actions in specific situations (Evans et al., 2016). This theory provides evidence to verify the heuristic process of a perceived affordance on outcomes. Thus, this study infers that the perceived explainability of algorithms may promote the possibilities for resistance against algorithms. Recent research also provides more empirical evidence about the view. Facebook users who understood the logic of algorithms trained the algorithms for better content curation, co-produced by both users and platforms (Eslami et al., 2016; Velkova and Kaun, 2021). One study found that co-production would enhance or constrain certain engagement practices (Gerlitz and Helmond, 2013). Understanding and explaining how algorithms work led to engagement with gearing algorithmic workings toward users’ benefits. Cotter (2019) found that content producers of Instagram, such as online celebrities, were trying to understand how recommendation algorithms work to stimulate or boost their own popularity. Another study suggested that perception and understanding of Twitter algorithms promoted users’ rights to achieve resistance goals (Treré et al., 2017). In some situations, perceived algorithms can promote tactical interventions to trick algorithms (Bucher, 2017). For ordinary users, privacy is an important right to be protected. In some cases, for example, when a privacy risk is perceived, user perception of explainability may constrain engagement practice and facilitate the tactic of obfuscation. Therefore, the present research hypothesizes:

*H4*: Perceived explainability of algorithms will have a positive effect on avoidant resistance (H4a); obfuscated resistance (H4b).

Affordance theory requires recognition of the role of affordances in mediating the link among objects (e.g., technology or some technological features), human goals, and outcomes (Parchoma, 2014; Evans et al., 2016). Ignoring this aspect of affordances implies that an object results in the behavioral outcome without any sign of the underlying process—a theoretical leap (Evans et al., 2016). Therefore, in the context of algorithms, the present study deduces that perceived algorithmic affordance mediates the relationship between motivations and resistance actions. These processes suggest the following hypotheses:

*H5*: Perceived explainability of algorithms mediates the relationship between entertainment and avoidant resistance (H5a); entertainment and obfuscated resistance (H5b).

*H6*: Perceived explainability of algorithms mediates the relationship between peeking and avoidant resistance (H6a); peeking and obfuscated resistance (H6b).

*H7*: Perceived explainability of algorithms mediates the relationship between escapism and avoidant resistance (H7a); escapism and obfuscated resistance (H7b).

**Figure 1:**
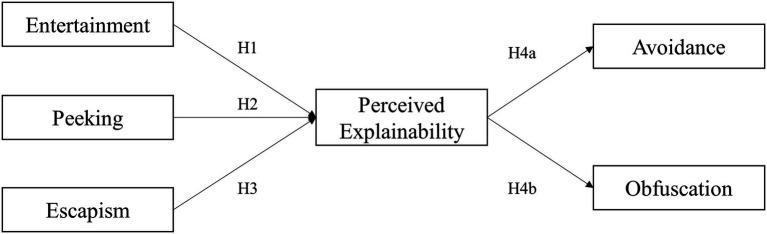
Conceptual model in the present study.

Figure 1 delineates the model used in the present study.

## Materials and methods

### Construction of the scale of algorithmic resistance

The study focused on the algorithm recommender systems of short video platform, a typical form of social media. The first reason was that algorithm recommender systems are the most common algorithm system. The second reason is that, compared with other platforms, the content presentation form of short video is easier for users to perceive the recursive relationship with the algorithm, which is conducive to the in-depth research.

Based on reviewing the previous literature, this study identified three subdimensions: productive resistance, avoidance, and obfuscation. For ordinary users, the “threshold” of productive resistance is too high to be widely popular. Thus, this study focused on the other two dimensions of resistance. And four focus group discussions (*n* = 16) were organized to construct effective items for the Algorithmic Resistance Scale (ARS). Participants were asked whether they would resist algorithmic recommendations in short video and what resistant behavior they would take. The participants included 16 doctoral students with professional knowledge of using algorithm services. This study collected a pool of 12 items relating to resistance. To improve the applicability of the scale among the public, 30 participants with no professional background (28 ≤ age ≤ 62, men = 14) were subsequently invited to test the items. They were asked to identify and exclude repetitive or puzzling items. The scale was distilled down to six items and was adapted to the expressions which were easy to understand for the public.

### Procedure and participants

Data collection was in three phases. First, to examine the structure of ARS, recognize the number of factors to be preserved, and eliminate the substandard items, a pre-survey (*n* = 500) was conducted for Exploratory Factor Analysis (EFA). Second, using the second pre-survey sample (*n* = 500), the study performed confirmatory factor analyses (CFA) to test the construct validity of the ARS and further decrease redundancy in the scale. Third, after the structural analyses were complete, the ARS was included in the formal questionnaire to investigate how people perceive algorithms and the behavioral outcomes.

All surveys were conducted *via* online questionnaires, which were then entrusted to a professional market research company named *Ipsos*. The agency used quota sampling, stratified by age, gender, and educational level of Chinese netizens. According to the 47th Statistical Report on China’s Internet Development, the number of short video users accounts for 88.3% of the total number of netizens in China, and the user composition is basically the same as that of the overall Internet users. The study makes a more accurate quota division combined with the user information disclosed in the data reports of the two main short video platforms (Douyin and Kuaishou). Data collection commenced in March 2021. Finally, the agency collected 2,000 effective samples. And the number of samples is also our requirement which should ensure at least 2,000 effective samples. This study concludes with three data analysis processes: EFA, CFA, and structural equation model. Referring to previous studies with similar procedures, it is found that the sample size of EFA and CFA is about 500, and the sample size of the overall model validation is about 2,000 (Bruun et al., 2016; Zarouali et al., 2021). Therefore, this study adopts a large sample size of 2000 to ensure its rationality. Participants were aged between 16 and 62 years (M = 31.36, SD = 9.67). The sample composition is presented in Table 1, which conforms with the basic composition of Chinese short video users.

**Table 1:** Participant composition (*N* = 2,000).

Variables	Levels	Frequency	Percentage (%)
Gender	Male	1,054	52.7
	Female	946	47.3
Education	Junior middle school and below	152	7.6
	High school	472	23.6
	Junior college	521	26.05
	Bachelor	782	39.1
	Master and above	73	3.65

### Measurement

#### Short video goals pursuit: Entertainment, peeking, and escapism

Scale items for short video users’ goals were developed from the Internet-use-motivation literature. Items suggested by Papacharissi and Rubin (2000) were used as the basis for the operationalization of the entertainment construct. This scale, containing three items, was modified based on the short video scenarios. For example, “Using short video is a way of entertainment,” “I use short video to relax,” and “Using short video is enjoyable.” The scales for peeking were adapted from Lee et al. (2015) and consisted of three items. For example, “To browse daily lives of celebrities,” “To browse daily lives of people from all walks of life,” and “To browse the lives of people with similar experiences.” Items to measure escapism were initially taken from Stenseng et al. (2012). Considering the motivations of negative behavior, the study particularly selected the dimension of the self-suppression scale, containing six items (e.g., “When I use short videos, I try to suppress my problems,” and “I try to prevent negative thoughts about myself.”). Entertainment and Peeking constructs were measured on a seven-point scale (1 = completely disagree, 7 = strongly agree), while participants rated the *Escapism Scale* items on a five-point scale.

#### Perceived explainability of algorithms

Items to measure Perceived Explainability were adapted from Shin (2021). The scale measured the users’ awareness and understanding of algorithm recommender systems of short video, comprising three items (e.g., “I found algorithms are easily understandable,” “I think the algorithm services are interpretable,” and “I can figure out how the platforms recommend content to me.”). These items were rated by participants on a seven-point scale (1 = do not agree at all, and 7 = completely agree).

#### Algorithmic resistance

The six items of ARS were developed and refined from the focus groups and two pretests were applied in this survey. The items of algorithmic resistance broadly assess the “dark side” of online participation (Lutz and Hoffmann, 2017), such as not actively generating interactive data with algorithms or interacting consciously and critically with algorithms (Gran et al., 2020; see Table 2 for the final scale items). Responses were recorded along a seven-point scale.

**Table 2:** Exploratory factor analyses (*n* = 500).

Items	1	2
When I’m aware of algorithms in short video platforms…		
AR1. I seldom actively train algorithm recommender systems.	**0.761**	0.189
AR2. I rarely choose what to browse actively.	**0.901**	0.052
AR3. Usually, I browse what the platform recommends to me.	**0.823**	0.214
OR1. I will regularly and consciously train algorithm recommender systems to make the content more diversified, instead of recommending only the content I like.	0.037	**0.829**
OR2. I will often consciously train algorithm recommender systems so that the platform cannot accurately grasp my preferences.	0.366	**0.755**
OR3. I will often consciously train algorithm recommender systems to obtain more content which I did not get before.	0.147	**0.878**

Note: Table 2 indicates the results of the rotating component matrix. And the bold values allow the readers to see the factor loadings more clearly.

### Data analysis

#### Exploratory factor analysis of algorithmic resistance

Using the exploratory sample (*n* = 500), the six resistant behavior items were subjected to statistical analyses to ensure item variance, determine the factor structure, and set up acceptable item-total correlation. Principal component analysis (PCA) with varimax rotation was performed to determine the underlying structure that exists for resistant behavior. The PCA was assessed using the following standards: eigenvalue (*q* > 1.0), variance explained by each component, loading the score for each factor (*q* ≥ 0.60), and the meaningfulness of each dimension. EFA (using SPSS version 26) found two factors with eigenvalues greater than 1.0. The item loadings were all above 0.75 (see Table 2). Thus, all six items remained. A meaningful two-component solution was obtained, with the two factors accounting for 72.268% of the total variance. The first factor, labeled “avoidant resistance,” explained 37.081% of the variance after rotation, and its three items formed a reliable scale, as evaluated by Cronbach’s alpha (*α* = 0.802). The second component, “obfuscated resistance,” consisted of three items, accounting for 35.187% of the variance (*α* = 0.794).

#### Confirmatory factor analysis

In the second stage of analysis, another pretest sample (*n* = 500) was used to do Confirmatory Factor Analyses (CFA) in Amos version 26. CFA was used to validate the results of the EFA. The findings indicate the acceptable factor loadings of items. The factor loadings for all were significant, which means valid internal consistency is good. To evaluate the validity, correlation analyses were carried out to calculate reciprocal relationships among variables. Pearson’s r was employed to assess the significance of observed relationships. There were no indications that the mutual relations among the variables are multicollinearity. Thus, discriminant validity was acceptable (see Table 3).

**Table 3:** Confirmatory factor analyses results (*n* = 500).

Construct	Items	Unstd.	S.E.	t-value	Std	CR	AVE
Avoidant resistance	AR1	1			0.676	0.812	0.593
	AR2	1.372	0.100	13.683^***^	0.872		
	AR3	1.197	0.086	13.908^***^	0.750		
Obfuscated resistance	OR1	1			0.674	0.821	0.609
	OR2	1.231	0.086	14.266^***^	0.747		
	OR3	1.418	0.102	13.948^***^	0.902		

*** *p* < 0.001.

The data from the factor loadings (unstandardized and standardized), composite reliability (CR), and AVE values for each construct suggest that the indicators account for a large portion of the variance of the corresponding latent construct and thus provide evidence for measurement modeling.

## Results

### Structural model testing

First, bivariate correlations were computed using all formal investigation samples (N = 2000). Descriptive statistics and the zero-correlations of all variables are displayed in Table 4. The results were credible because all the internal consistency alphas values exceeded 0.75. Users evaluated entertainment highly (M = 5.77, SD = 1.20), and evaluation of entertainment correlated positively with the perceived explainability (*r* = 0.279, *p* < 0.01). Peeking and escapism also correlated positively with the perceived explainability (*r_peeking_* = 0.508, *r_escapism_* = 0.334, *p* < 0.01). The findings indicated that the perceived explainability was positively related with avoidant resistance (*r* = 0.229, *p* < 0.01) and obfuscated resistance (*r* = 0.467, *p* < 0.01).

**Table 4:** Descriptive statistics, alpha coefficients, and correlations (*N* = 2,000).

	M	SD	1	2	3	4	5	6
1. Entertainment	5.77	1.20	(0.892)					
2. Peeking	5.02	1.14	0.491^**^	(0.776)				
3. Escapism	3.34	0.85	0.144^**^	0.362^**^	(0.845)			
4. Perceived explainability	4.84	1.20	0.279^**^	0.508^**^	0.334^**^	(0.826)		
5. Avoidant resistance	4.47	1.34	0.079^**^	0.289^**^	0.341^**^	0.229^**^	(0.802)	
6. Obfuscated resistance	4.60	1.29	0.064^**^	0.412^**^	0.406^**^	0.467^**^	0.415^**^	(0.800)

Internal reliabilities (alpha coefficients) for the constructs are given in parentheses on the diagonal.

** *p <* 0.01;

This study drew on the bootstrapping method (Preacher and Hayes, 2008), which produces 95% bias-corrected confidence intervals from 5,000 resamples of the data. And goodness-of-fit indices were used to evaluate the model whether it could be accepted or not. Previous research used the chi-squared value per degrees of freedom (χ2/df), Comparative fit index (CFI), Goodness of fit index (GFI), and the root mean square error of approximation (RMSEA) to measure the model fit (Jackson et al., 2009). In this study, most of the indices indicated a good fit suggesting a high probability of good fit (see Table 5).

**Table 5:** Model fit indices.

Fit statistics	Model	Suggested value
χ^2^/df	9.960	<3
RMSEA	0.067	<0.08
CFI	0.915	>0.9
GFI	0.908	>0.9
AGFI	0.883	>0.8

**Figure 2:**
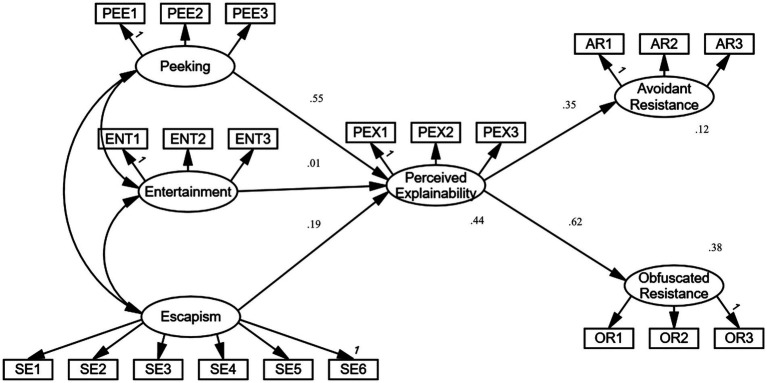
Structural equation model with results of the AMOS analysis (*N* = 2,000).

The results of structural path testing supported most of the hypotheses (Figure 2; Table 6). All the path coefficients were statistically significant (*p* < 0.001) except the path from entertainment to perceived explainability (*p* = 0.639), meaning H1 was not supported. Perceived explainability is significantly influenced by the motivations of peeking and escapism, determined by peeking. These factors altogether account for 44% of perceived explainability variable (R^2^ = 0.44). Obfuscated resistance values are greatly influenced by the perceived explainability. The model explained a significant portion of the variance in each construct. The strong paths imply a fundamental connection between the perceived algorithmic affordance and its antecedents. Given the significant effect of the perceived algorithmic affordance on avoidant resistance and obfuscated resistance, it would be desirable to examine the possible mediating effects of perceived algorithmic affordance on other outcomes.

**Table 6:** Path results.

Paths	Coefficient	S.E.	C.R.	Inference
H1: Entertainment → perceived explainability	0.012	0.025	0.469	Rejected
H2: Peeking → perceived explainability	0.516	0.033	15.498^***^	Supported
H3: Escapism → perceived explainability	0.259	0.036	7.131^***^	Supported
H4a: Perceived explainability → avoidant resistance	0.335	0.027	12.417^***^	Supported
H4b: Perceived explainability → obfuscated resistance	0.577	0.028	20.819^***^	Supported

*** *p <* 0.001.

### Test for mediation

There was also evidence of a significant indirect effect of the motivations of peeking and escapism *via* perceived explainability on avoidant and obfuscated resistance. Specifically, the motivations of peeking and escapism were both indirectly associated with increased avoidant resistance *via* perceived explainability (*ß*_Peeking_ = 0.190, *p* < 0.001, CI95% [0.145, 0.240]; *ß*_escapism_ = 0.067, CI95% [0.043, 0.100]). Therefore, H6a and H7a were supported. Regarding obfuscated resistance, there was evidence of a significant, positive indirect effect such that the motivations of peeking and escapism were associated with obfuscated resistance, respectively, (*ß*_Peeking_ = 0.339, *p* < 0.001, CI95% [0.280, 0.403]; *ß*_escapism_ = 0.119, CI95% [0.081, 0.164]), supporting H6b and H7b. The results indicated that the indirect effect of entertainment *via* perceived explainability is not significant. Therefore, H5a and H5b were not supported.

## Discussion

This study explains the formation progress of users’ algorithmic resistance with empirical evidence, which is hardly explored by previous research. Specifically, the motivations of peeking and escapism positively correlate with the perceived explainability of algorithms, which, in turn, encourages avoidant and obfuscated resistance. These results supported H1–H4, and H6–H7. In line with previous research on algorithmic awareness and behavior, the findings strengthen the active role of users (Belanche et al., 2017b, 2019; Dogruel et al., 2020). The good use of algorithms is not only reflected in achieving personal or organizational goals but also reflected in avoiding risks. As Taylor’s view, to ensure data fairness, users should have the freedom to access data technology and the freedom to refuse (Taylor, 2017). Besides, this study believes that users should have the ability to refuse.

Different from productive resistance, the two tactics of algorithmic resistance concerned in this paper are not destructive. The user achieves the goal of resistance based on the algorithmic rule. Like skipping advertisements (Belanche et al., 2019), deliberately not interacting with the platform is an avoidance behavior. Intentional non-participation behavior reflects the subjectivity of the user. Algorithms will evoke some sense of identity in the user (Karizat et al., 2021). When this awareness makes users feel uneasy, they may adjust their strategies.

Different from previous studies, the findings imply that users’ subjectivity is also affected by the functions of technology, context, and personal goals. For the purpose of economic interests, bloggers will obey the algorithm rules to improve their visibility, but they are also controlled by the algorithm rules (Mac Donald, 2021). For the purpose of peeking and escapism, when users realize algorithms accurately evaluate them, they become more sensitive and vigilant about algorithms (Bucher, 2017).

Algorithms may ultimately define the scope of human knowledge and cognitive style (Gillespie, 2014). The algorithm determines the content people are exposed to and affects the way users perceive the world (Just and Latzer, 2017). Its power and prejudice may pose a threat to personal rights and publicity (Nguyen et al., 2014). The ability to develop algorithmic resistance may benefit people’s public life and democracy.

### Theoretical implications

The present study offers several theoretical implications and complements existing research. First, this study offered a new theoretical perspective on the interaction between users and algorithms by employing the affordance framework. Technological determinism and constructivist views are limited in explaining technology use (Leonardi and Barley, 2008). Affordance perspectives break through the limitation of one explanatory power by highlighting the multiple relations between technologies, users, and the context. *A priori* knowledge of algorithms is not necessarily required to perceive algorithmic affordance, which can emerge and accumulate through experimentation and adaptation with algorithms. Thus, the concept of perceived algorithmic affordance is a result of the interaction and the cognitive basis for triggering subsequent interactions. The model in this study shows a cross-section of interactions between users and algorithms. We can regard the actual reciprocal actions as the process of circulation of the model. Specifically, users’ needs have promoted the tactics through perceived algorithmic affordance. This interactive experience may change the prior understanding of how algorithms work, triggering a new interaction in a different context. Therefore, our findings provide a framework ideally suited to explain the recursive loops of users and algorithms.

Second, our work highlights the significance of the specific context on perceived affordance. Most studies have mentioned outcomes of perceived affordance but ignored their predictors (Bucher, 2017; Shin, 2020, 2021). The present study provided an empirical context for interacting with algorithms to make affordance—a type of middle-range theorizing—concrete and contextual. Our findings contributed to developing the specific predictors of perceived affordance *via* discussion of contextual factors. In the present study, peeking, escapism, and entertainment reflect the needs and goals of users, forming different contexts combined with social media practice. The findings show how diverse motivations have different effects on perceived algorithmic affordance. The latter could also be regarded as an ability for increased awareness and understanding of how algorithms function, corresponding to a new digital divide (Gran et al., 2020; Hargittai et al., 2020). As algorithms increasingly influence content delivery and presentation, it is important to determine whether understanding algorithms impacts our perceptions of the world and our actions in a decisive way. This information may have implications for related issues of public life and democracy (Dogruel et al., 2020; Gran et al., 2020). Hence, to narrow the gap of perceived algorithmic affordance among users, more scholarly attention should be paid to the specific context.

Last, our study extends the possible outcomes of algorithmic affordance. Former studies show that the understanding of algorithm operation could promote algorithmic subjection, for instance, obeying the algorithmic logic to enhance the visibility of content (Bucher, 2017; Karakayali et al., 2018). However, few studies explore the algorithmic resistance among ordinary users. Some scholars claim that the variations in an affordance may relate to different outcomes, even contradictory behavior (Fayard and Weeks, 2014; Evans et al., 2016). Thus, our findings supplement empirical evidence to support how perceived algorithmic affordance might trigger the behavioral outcomes of resistance. Our work pays attention to two types of resistance from the list of possible resistance tactics: avoidant resistance and obfuscated resistance.

The similarity of the two outcomes may be that they are both tactics of resistance rooted in the socio-cultural context of China. As stressed earlier by Gibson (1979), only considering users with specific needs and practices within a particular socio-culture context can make the discussion on affordance more significant. Thus, this study set out to explore algorithmic affordance and its impact on resistant behavior in the socio-cultural context of China. Individuals in this context tend to attach importance to social harmony and implicit, indirect expressions (Hall and Hall, 1989). In this socio-cultural situation, users’ perception of algorithmic affordance might incline to “moderate” resistance, such as avoidance and obfuscation, rather than antagonistic conflict (Hofstede et al., 2005). However, the difference between the two outcomes lies in whether data are actively produced. Avoidant resistance emphasizes minimizing the production of behavioral data, while obfuscation stresses creating misleading, false, or ambiguous data and feeding it back to the algorithms (Casemajor et al., 2015). Unlike features, affordances are not binary, but somewhat variable. Scholars suggest this may relate to individual differences, such as demographics, motivational traits, goals, experience, and capabilities (Lai, 2019). The variability of affordances might promote the different behaviors of individuals using the same technology to achieve specific outcomes (Evans et al., 2016). Our findings merely show the two resistant outcomes of perceived algorithmic affordance, rather than deeply explaining the specific mechanisms leading to particular outcomes. Thus, we recommend that future studies should explore a wider breadth of outcomes of perceived algorithmic affordance and elaborate how the variations of affordance may lead to the outcomes.

### Practical implications

The study outlines the game between ordinary users and algorithms embedded in social media platforms. The process of coevolution has remarkable practical implications for users, algorithms, and the industry. First, the findings imply that users will have greater autonomy in content curation as their understanding of algorithms grows. As an infrastructural technology embedded in our daily life, algorithms may influence users’ options, but they do not determine them. Although recommendation algorithms are designed to direct users toward particular modes of engagement with the platforms, it is impossible to fully predict the ways in which users might use algorithms. Users may find more meanings and functions than intended by the designers. The algorithmic resistance illustrated in the study is exactly the strategy selection for users to game with algorithms. Users who perceived algorithmic affordance could resist algorithms by reshaping the datasets on which the algorithm crafts its output. Through the process, users and algorithms could coordinate the content curation. Overall, realizing and perceiving more about the structural power that shapes the platforms is an online skill and a necessary condition for managing information as a citizen.

Second, the synergy between users and algorithms may optimize algorithms and related services. Based on the research model, we could infer that these restricted conditions may reveal that algorithmic resistance lacks sustainability. In other words, the interaction between users and algorithms suggests a dynamic interplay between resistance and subjection. However, for the platforms, the resistance of avoidance and obfuscation are not conducive to platform owners’ development of new modes of profitable subjection to gain a competitive advantage. From the platforms’ perspective, the work of designers is to narrow the gap in perceived and actual affordance (Norman, 1993; Fayard and Weeks, 2014). Algorithmic “bugs” will eventually be modified by their designers, ensuring that algorithms will co-evolve with use and resistance. On the other hand, the platform can further improve users’ awareness and knowledge formation of algorithms by improving the visibility of information and functions of algorithms, optimizing the interface, and promoting the harmonious coexistence and collaborative development of users and algorithms.

## Limitations and future studies

To provide a reasonable understanding of the applications of this study, its limitations should be set out. First, the research design has inherent limitations as self-reporting measures may be subject to bias, and participants’ perceptions are subjective. Researchers do not necessarily know the ground truth of algorithms, making it difficult to develop measures of how the algorithms function (DeVito et al., 2018; Hargittai et al., 2020). Thus, the relevant empirical research is very limited. While algorithms certainly constitute a black box (Pasquale, 2015), we should not remain at the stage of theory and conjecture. This present study focused on a more general perception of algorithmic affordance, rather than a particular algorithmic operational rule. The study used a self-reporting scale to measure perceived algorithmic affordance. Thus, the challenge of using objective measures to understand how algorithms work was overcome. Although self-reporting scales have some limitations, they also provide the most direct and cost–benefit methods for measuring media-related concepts in large samples (Slater, 2004). Further study needs to use objective assessment for crosschecking the validity of self-report measure. Moreover, to avoid the limitations of one certain method, studies can employ multiple methods such as natural experiments, interviews, and questionnaires to deepen the study of the interactions between users and algorithms.

Second, the cross-section survey-based methodology did not lend itself to reaching causal conclusions. To gain a more precise understanding of recursive loops between users and algorithms, studies could adopt experimental designs with a longitudinal or panel study approach—to track repeated observations of users over long periods—and measure the interaction effects between the users and AI algorithms.

Third, the study only considered the context of recommendation algorithms system in short video platforms—just one kind of algorithmic situation. Another significant algorithmic context is algorithmic decision-making (Kitchin, 2017; Danaher, 2019), embedded in daily life as intelligent assistants. Our model suggests that users may decide to resist algorithms. Algorithmic decision-making is more complex than recommendation algorithms, depending more on the context, experience, and autonomy (Danaher, 2019; Dogruel et al., 2020). Thus, future studies could expand the model to other situational factors to examine how user experience and autonomy impact algorithmic tactics.

Finally, this study considered the socio-cultural context of China, but did not explore the impact of the cultural milieu on perceived algorithmic affordance and related actor strategies in the model. Further research could explore the interaction of users and algorithms using a comparative, cross-cultural perspective in more detail. For example, future studies could compare the differences between the users’ perceived algorithms and behavioral outcomes in Douyin and TikTok (Chinese and international versions of the same application) to analyze the role of culture.

## Conclusion

This study considered why and how people resist algorithms in the context of short videos. Based on the perspective of affordance, we uncovered the formation mechanism and heuristic process of algorithmic resistance. The findings show that different goals will mobilize users’ perceptions of algorithms and trigger various strategies. To avoid being tracked by algorithms, users may choose tactics of algorithmic resistance. The present study indicated that the motivations of short videos are positively related to algorithmic resistance through the key mediating role of perceived algorithmic affordance. The study expands and enriches the theoretical framework of affordance by revealing new predictors of perceived algorithmic affordance and the possible outcomes of algorithmic resistance. By focusing on algorithmic resistance possibilities, the current research provides insights into the synergy between users and algorithms to co-evolve and create reality. As algorithms gradually become more extensively embedded in all aspects of human social life, we need more empirical research representing the user perspective about the recursive loops between users and algorithms.

## Data availability statement

The datasets presented in this article are not readily available because they are part of an ongoing research project. Requests to access the datasets should be directed to baiqiyu_pku@163.com.

## Ethics statement

The studies involving human participants were reviewed and approved by New Media School, Peking University. Written informed consent for participation was not required for this study in accordance with the national legislation and the institutional requirements.

## Author contributions

XX and YD contributed to the design, conceptualization, methodology, data analysis, and writing. QB contributed to the design, revision, and supervision of the work. All authors contributed to the article and approved the submitted version.

## Funding

This research was supported by the National Social Science Fund of China (grant number 18ZDA317).

## Conflict of interest

The authors declare that the research was conducted in the absence of any commercial or financial relationships that could be construed as a potential conflict of interest.

## Publisher’s note

All claims expressed in this article are solely those of the authors and do not necessarily represent those of their affiliated organizations, or those of the publisher, the editors and the reviewers. Any product that may be evaluated in this article, or claim that may be made by its manufacturer, is not guaranteed or endorsed by the publisher.
